# Retinal pigment epithelium and age‐related macular degeneration: A review of major disease mechanisms

**DOI:** 10.1111/ceo.13834

**Published:** 2020-08-17

**Authors:** Shreya Somasundaran, Ian J. Constable, Carla B. Mellough, Livia S. Carvalho

**Affiliations:** ^1^ Centre for Ophthalmology and Visual Science/Lions Eye Institute University of Western Australia Nedlands Western Australia Australia

**Keywords:** age‐related macular degeneration, apoptosis, eye, necrosis, retinal pigment epithelium

## Abstract

Age‐related macular degeneration (AMD) is a progressive degenerative disease that is the leading cause of vision loss in the elderly population. Degeneration/dysregulation of the retinal pigment epithelium (RPE), a supportive monolayer of cells underlying the photoreceptors, is commonly seen in patients with AMD. While treatment exists for the neovascular/wet form of AMD, there is currently no cure for the non‐exudative/dry form of AMD, making it imperative to understand the pathogenesis of this disease. Although our understanding of the aetiology of AMD has increased over the years, the underlying disease mechanism has not yet been identified, mainly due to the multifactorial nature of this disease. Herein, we review some of the commonly proposed degeneration pathways of RPE cells and their role in the pathogenesis of AMD; including activation of the complement cascade, oxidative stress‐induced cell death mechanisms, dysfunctional mitochondria and the role of crystallins in AMD disease progression.

## 1 INTRODUCTION TO AGE‐RELATED MACULAR DEGENERATION

Age‐related macular degeneration (AMD) is the leading cause of bilateral central vision loss in the elderly population in developed countries.^1^ The macula is the central region of the retina that is responsible for sharp, photopic vision. The fovea lies at the centre of the macula and has the highest density of cone photoreceptor cells in the retina.^2^ Thus, degeneration of this area can have a profound impact upon visual acuity, as well as the physical and mental wellbeing of the individual.

Damage to the retinal pigment epithelium (RPE) is considered to be a hallmark of AMD. There are several changes to the RPE and its surrounding microenvironment; however, some of these changes also occur over the course of normal ageing in the eye. One of the most noticeable changes is a decrease in the number of RPE cells, although this loss is relatively low compared to RPE loss in patients with AMD.^3^ RPE pigmentation and shape also change during ageing, as the number of melanosomes decrease and lipofuscin granules accumulate.^4^ As a result, there is photoreceptor cell loss and thickening of the Bruch's membrane, which leads to loss of elasticity of the Bruch's‐choroid complex.^5^ However, it is when these changes are exacerbated and occur abnormally that disease progression ensues.^6^

The RPE is a highly active tissue due to the tightly regulated support it offers to the neural retina.^7^ Some important roles of RPE include the daily phagocytosis of photoreceptor outer segments (POS), secretion of neutrophic factors required to stabilize the neural retina and scavenging for damaged ROS.^7^, ^8^ While photoreceptors take part in the phototransduction cascade through isomerisation of 11‐*cis* retinal to all‐*trans* retinal, the process of recycling all‐*trans* retinal back to 11‐*cis* retinal is supported by the RPE, making RPE an integral part of the visual cycle.^7^ Additionally, a single RPE cell interacts with several photoreceptors, making the RPE a highly metabolically active tissue.^8^

The AMD spectrum spans early‐stage AMD, during which drusen, pigment epithelial detachment (PED) and vitelliform lesions are observed, to late stage AMD, with choroidal neovascularization (CNV, neovascular AMD) or geographic atrophy (GA). Clinically, patients with early‐stage AMD commonly present with drusen (lipid) deposits in the space between the basal lamina of the RPE and Bruch's membrane, the innermost layer of the choroid. Occasionally soft drusen deposits, which lead to a higher risk of visual loss than hard drusen, observed during the early stages of AMD may progress to form large drusenoid PEDs. Deposits in the subretinal space (between the neural retina and RPE) called vitelliform lesions may also develop, due to the accrual of photoreceptor outer segment debris.^9^ Formation of both PED and vitelliform lesions are usually followed by the gradual formation of GA, which consists of atrophy of the outer retinal tissue, retinal pigment epithelium (RPE) and choriocapillaris in the macular region. More commonly, however, GA develops from calcification of soft drusen or de novo from reticular pseudodrusen or drusenoid deposits above the level of the RPE.^10^ In a colour fundus image, GA is typically characterized by atrophic hypopigmented lesions of the outer retina indicating the progressive loss of RPE, photoreceptors and underlying choriocapillaris.^11^ Atrophic lesions commonly arise from calcified drusen or de novo regions of reticular pseudodrusen around the fovea. Initially, GA may appear around the central macula, sparing the fovea.^12^ GA size progressively enlarges at a rate of 1‐2 mm^2^/year and expands concentrically to coalesce with other patches of GA, eventually eroding into the foveal centre.^10^ GA affects an approximate five million people globally,^10^ including 22% of people over 90 years of age,^13^ and these figures are set to rise with the projected increase in the ageing population. Although, given its complexity, the exact pathogenesis of GA is yet to be elucidated, advances in understanding the pathophysiological mechanisms underlying AMD have implicated several pathways. Interestingly, GA can occur in isolation or in combination with neovascular AMD. GA is the most damaging end point of the drusen life cycle, whereby these deposits appear in a cycle of formation and regression.^6^

CNV is associated with a more severe and rapid form of vision loss. Ten percent to 15% of all early‐stage AMD patients progress to develop CNV.^14^ CNV is defined by the abnormal growth of blood vessels under the retina, which often leads to exudation of fluid and/or blood into the subretinal space, the neural retina itself or the sub‐RPE space.^15^ Therefore, patients with CNV usually present with subretinal or intraretinal buildup of fluid. Overproduction of vascular endothelial growth factor (VEGF) is a key feature of CNV and current treatment options seek to inhibit the excess production of this protein.^9^ Hypoxia, inflammation and oxidative stress‐induced activation of the transcription factor hypoxia‐inducible factor‐1 are found in the retina of patients that present with CNV.^16^ Age‐related structural abnormalities within Bruch's membrane have also been associated with AMD. Damage to Bruch's membrane could make this structure more susceptible to the ingrowth of choroidal blood vessels from the choriocapillaris, the inner most vascular layer of the choroid that supplies the outer retina.^17^

AMD is a multifactorial disease with an unclear aetiology; however, age is the most consistent risk factor. Other factors that have been implicated include inherited genetic variations, oxidative stress, ethnicity, as well as environmental and lifestyle risk factors including smoking, diet and hypertension.^14^ Clinical studies have shown that a diet rich in omega‐3 fatty acids, vitamins, zinc supplements and anti‐oxidants could offer some protection against AMD.^18^ While amelioration of symptoms and visual loss can be achieved in patients with CNV through the use of anti‐angiogenic antibodies including ranibizumab, aflibercept and bevacizumab, the dry form of AMD remains untreatable, and the underlying pathophysiological mechanisms are still not well‐understood.^19^ This review will cover some of the key mechanisms proposed in instigating the development of AMD, in particular dry AMD, with a focus on RPE cell biology. It will discuss a broad spectrum of mechanisms ranging from widely accepted theories such as complement activation and oxidative stress‐induced cell death, to emerging theories such as dysfunctional mitochondria and the role of crystallins in disease progression.

## 2 ROLE OF COMPLEMENT CASCADE IN AMD

The complement system is part of the innate immune system and is necessary to prevent immune over‐activation and inflammation in tissues. Since the eye is a relatively immune‐privileged organ, the complement cascade is tightly regulated and its components are found only in low levels in the eye, with the major sources being the RPE and retinal microglia.^20^ Therefore, a number of diseases, including age‐related eye diseases such as AMD, might arise when the local immune homeostasis in the eye is disrupted.^20^ The role of this system in AMD has been suggested as a central driver in AMD pathogenesis, and there are multiple papers discussing its involvement.^20^, ^21^, ^22^, ^23^

The activation of the complement system is initiated by an insult that triggers the cell to release signals such as cytokines, chemokines and growth factors to neighbouring cells. This elicits pathophysiological responses associated with the clearing of microbes and damaged cells, an attack of pathogen membranes, and the promotion of inflammation.^20^ The complement system assists the innate immune system in clearing pathogens and convertase enzymes play a central role in complement activation. The system can be activated through the classical pathway (CP), the mannose‐binding lectin pathway and the alternative pathway. All three pathways converge at the activation of complement component 3 (C3) convertase to cleave the C3 complex and subsequent downstream activation of the complement component 5 (C5) convertase to cleave the C5 complex.^22^ The resulting complex C5bC9, or membrane‐associated complex (MAC) is crucial in the process of lysis and promotion of inflammation. Similarly, complement fragments of the cascade such as the production of C3a during the cleavage of the C3 complex and C5a during the cleavage of the C5 complex, are essential anaphylatoxins that assist in anaphylaxis, chemotaxis and immune regulation. C3b assists in opsonisation, a process that facilitates the stronger attraction of molecules, microbes or apoptotic cells to the surface receptors of immune cells, and therefore promotes phagocytosis of antigens and apoptotic cells.^20^, ^21^ Under normal physiological conditions, MAC is safely degraded by S‐protein/vitronectin,^20^ thereby maintaining homeostasis.

Dysregulation of this tightly controlled system can lead to an increase in complement activation and increased complement turnover at the choriocapillaris, preceding AMD.^24^, ^25^ After a hypothesis by Johnson et al on the potential of the immune complex to be involved in the formation of drusen, immunocytochemical evidence showed components of the complement system,^26^ including C3, C5 and C9, to be present in drusen of AMD patients.^27^ Additionally, many studies show that drusen deposits are composed of lipids, proteins and complement products, strongly pointing towards the involvement of an overactive complement system in disease pathogenesis.^28^, ^29^, ^30^ Complement C3, complement factor F, complement factor H (CFH) and MAC have been identified in drusen and AMD lesions.^28^ Plasma levels of C3, C3d, Bb and C5a were also increased in patients with AMD.^31^, ^32^ However, elevated complement components in both plasma and near AMD lesion brings into question whether the impaired complement turnover is a systemic or local effect.^21^

GA has been hypothesized to arise primarily due to drusen deposits disrupting the flow of nutrients and clearance of waste products between the choroicapillaris and RPE. This disruption leads to cell death that typically occurs in patches, as observed in the clinical presentation of GA.^21^ Although neovascular AMD is not known to arise due to an impaired complement system, interestingly, inhibition of C3a, C5a, complement factor B (CFB) and MAC has been shown to supress CNV in a laser‐induced mouse model.^30^, ^33^

While age, diet and lifestyle are shown to play an important role in the development of AMD, genetic variants in the complement system can heavily predispose an individual to the risk of developing AMD.^34^ In 2005, three independent studies stated that chromosomal region 1q31, which encodes for CFH, is a major susceptibility locus for AMD. At other chromosomal locations, polymorphisms in CFB, C2, serpin peptidase inhibitor clade G member 1 (a complement component 1 inhibitor) and C3 have also shown to increase the risk of developing AMD.^35^ The HtrA serine peptidase 1 (*HTRA1*) gene encoding the HTRA1 protein located in chromosome 10q26, in the region of the age‐related maculopathy susceptibility 2 (*ARMS2*) gene, activates both the alternative and calssical pathways. This risk variant has been commonly associated with AMD^35^ and increases actin‐binding protein production by 2‐fold.^36^ However, deletion of CFH‐related genes 1 and 3 (*CFHR1* and *CFHR3*) is proposed to be protective in AMD.^37^ This may be interesting to explore for potential therapeutic benefits.

There are several drugs in various phases of clinical trials that target specific complement components in AMD, including C3, C5 and complement factor D. These complement‐based therapeutics inhibit the activation of the complement system at various points. While lampalizumab, that binds to complement factor D, was one of the first antibodies to show experimental therapeutic effects, it was not effective in reducing the mean size of GA lesions.^38^ Many of the ongoing clinical trials undertaking a complement system modulation approach have shown modest therapeutic effects, mostly attributed to the late stage of therapeutic intervention, issues with dosing between individuals and insufficient drug delivery. Studies show that it might be useful to target the complement system during the earlier stages of AMD, before progression into GA or CNV.^39^ More information on complement‐based clinical trials and their outcomes can be found in the comprehensive review by Wu and Sun.^38^

With the increasing amount of literature exploring the involvement of the complement cascade in AMD, it is clear that any disruption to the immunological stability of the eye is potentially detrimental. Although there is strong evidence pointing towards a prominent role of complement in AMD, it is equally important to consider other disease mechanisms to fully understand what may be happening during AMD disease progression.

## 3 RPE CELL DEATH PATHWAYS IN AMD: NECROSIS, APOPTOSIS OR PYROPTOSIS?

**FIGURE 1:**
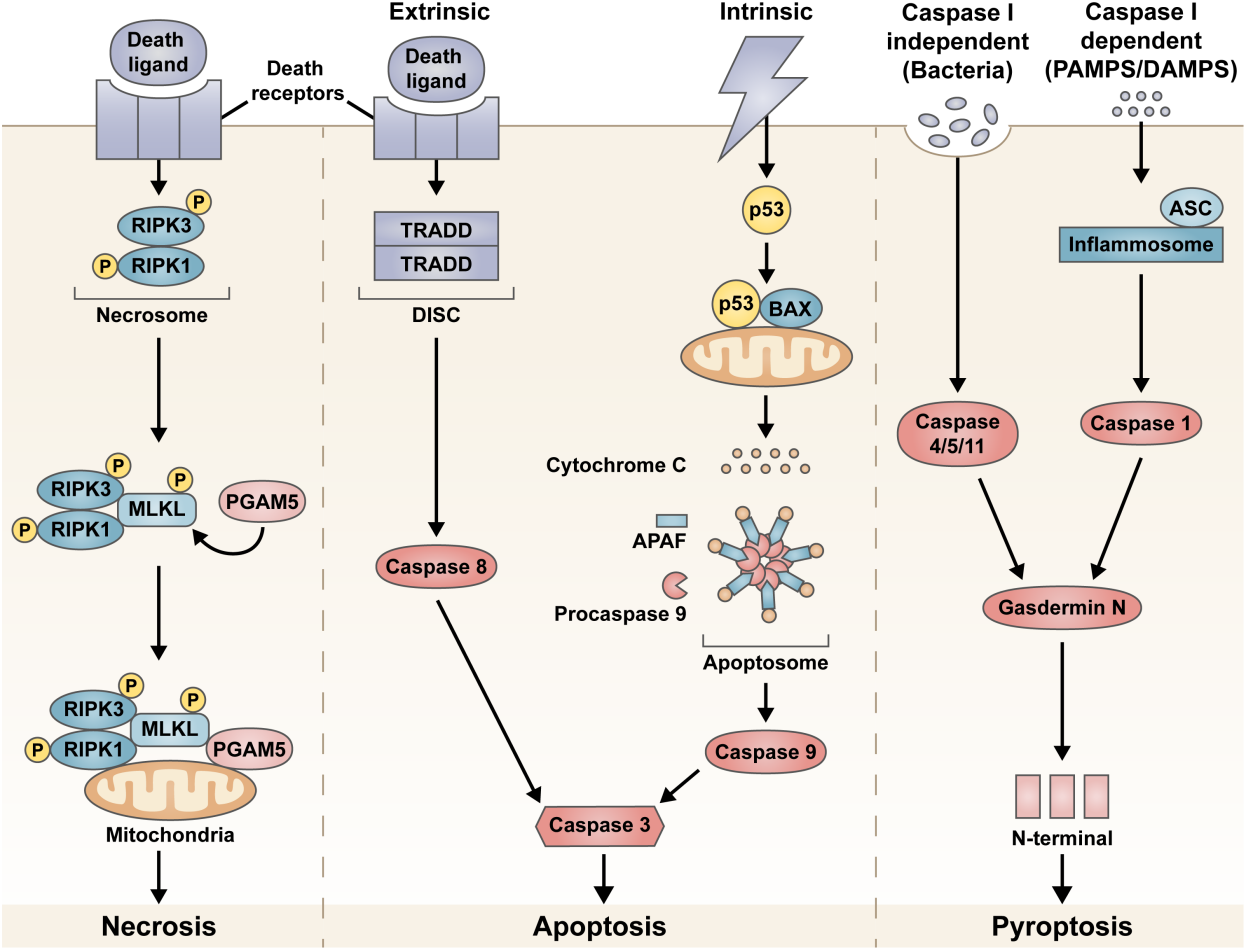
Overview of the cell death pathways of necrosis, apoptosis and pyroptosis proposed as the retinal pigment epithelium (RPE) cell death mechanism in age‐related macular degeneration (AMD). In necrosis, the necrosome forms a complex with other proteins and attaches to the mitochondrial membrane initiating cell death. Programmed cell death can be triggered using either the intrinsic or extrinsic pathways, which eventually culminates in the activation of caspase 3 and cell death via apoptosis. Pyroptosis can be activated in either caspase 1 dependent or independent manner. Both pathways lead to the generation of a N‐terminal fragment that triggers cell death

It is widely accepted that photoreceptors die through apoptosis in AMD.^5^ Although early studies had shown that RPE cells subjected to oxidative stress were shown to undergo apoptosis during early phases of AMD,^40^ it was not until chemical alteration of the extracellular matrix proteins and ultrastructural pathology studies were conducted, that both necrosis and pyroptosis were considered as potential mechanisms of RPE cell death in AMD patients.^41^, ^42^ This section will briefly explore the proposed cell death pathways of necrosis, apoptosis and pyroptosis in an attempt to better understand the mechanisms involved in AMD‐related RPE degeneration. A schematic overview of the different pathways discussed below is shown in Figure 1. More information about these cell death pathways can be found in reviews by Hanus et al and Man et al.^43^, ^44^

### 3.1 Necrosis

Necrosis is considered an unregulated form of cell death, which is characterized by cell swelling, cytoplasmic vacuole and bleb formation, energy depletion, lipid membrane disruption and loss of ion pumps or channels. The necrosis pathway is activated when the tumour‐necrosis factor (TNF) ligand binds to membrane death receptors (of the TNF family) and trimerizes. The pathway is largely mediated by receptor interacting protein kinases (RIPK). In the absence of caspase 8, RIPK1 and RIPK3 autophosphorylate to form a necrosome.^43^ The necrosome phosphorylates mixed lineage kinase domain‐like (MLKL) and recruits phosphoglycerate mutase 5 (PGAM5). The complex then attaches to the mitochondrial membrane to activate dynamin‐related protein 1 (Drp1) leading to fission and cell death.^43^

There are some studies that suggest the potential involvement of the necrotic cell death pathway in RPE death.^45^, ^46^ Hanus et al reported cardinal features of necrosis, such as ATP depletion and RIP3 aggregation, developing in ARPE‐19 cells treated with H_2_O_2_ or tert‐butyl hydroperoxide (tBHP) in order to induce oxidative stress.^45^ It was also reported that oxidative stress‐induced RPE death was largely prevented when RIPK3 was silenced.^45^ Similarly, a study by Li et al showed that oxidative stress‐induced RPE cells showed morphological similarities to necrosis, including cell swelling and loss of cell membrane integrity.^46^ However, further research exploring both in vitro and in vivo models is required to better understand the involvement of necrosis as a mechanism of RPE cell death in AMD.

### 3.2 Apoptosis

Apoptosis is a process of programmed cell death, which is characterized by caspase activation, DNA and nuclear fragmentation, mitochondrial outer membrane and lysosomal membrane permeabilisation, formation of apoptotic bodies and shrinkage of the cytoplasm.^43^ Apoptosis does not elicit an immunological response but rather inhibits inflammation, and any generated cell debris is phagocytosed by macrophages or other phagocytic cell types.^47^ Based on the type of cellular insult, either the *intrinsic* or *extrinsic* apoptotic pathways can be activated to trigger cell death, where the *intrinsic* pathway involves the mitochondria, and the *extrinsic* pathway involves cell membrane receptors.

Stressors such as UV or gamma radiation, increased reactive oxidation species (ROS) levels or viral infection can activate the intrinsic pathway.^48^ These cellular insults cause severe DNA damage, which leads to the inhibition of anti‐apoptotic factors and release of pro‐apoptotic factors such as Bcl‐2‐associated X protein (Bax). The activation of Bax causes the mitochondria to release cytochrome c into the cytoplasm. In the presence of ATP, cytochrome c binds to apoptotic protease activating factor 1 (Apaf1) to create a complex known as the apoptosome. The apoptosome activates procaspase 9 and subsequently caspase 9. Caspase 9 activates caspase 3 to trigger apoptosis of the cell.^48^, ^49^ The extrinsic pathway is activated when the TNF ligand binds to membrane death receptors (of the TNF family) and trimerizes. The receptors activate TNF‐receptor 1‐associated death domain (TRADD) and Fas‐associated death domain (FADD) to form a death‐inducing signalling complex (DISC). DISC recruits and activates caspase 8, which then activates caspase 3 to initiate apoptosis.^43^, ^48^

Several studies have shown the involvement of apoptosis in RPE degeneration.^50^, ^51^, ^52^, ^53^ Kaneko et al reported the activation of caspase 3 in the RPE of post‐mortem eyes with GA, with no caspase 3 detected in controls.^50^ Sharma et al have shown that 4‐hydroxynonenal (HNE)‐activated caspase 3 and p53 drive apoptosis in cultured RPE cells.^51^ In another study, primary RPE isolated from superoxide dismutase 2 (*SOD2*) knockout mice was subjected to oxidative stress and results showed terminal deoxynucleotidyl transferase dUTP nick end labelling (TUNEL) staining of cell death via apoptosis and cytochrome c leakage from the mitochondria.^52^ Ho et al found that c‐Jun N‐terminal kinases (JNK) or p38 inhibitors prevented the translocation of Bax to the mitochondria when oxidative stress was induced with H_2_O_2_.^54^A recent study has shown the activation of both apoptosis and pyroptosis pathways when RPE cells were stressed with exposure to a protein complex that initiates inflammatory cell death, the nod‐like receptor family pyrin domain containing 3 (NLRP3) inflammasome.^53^

### 3.3 Pyroptosis

Pyroptosis is an inflammatory method of programmed cell death. Pyroptosis is characterized by cell swelling, cell membrane rupture, chromatin condensation and loss of mitochondrial membrane potential.^44^ This cell death pathway can be both independent of, and dependent on, caspase 1. In a caspase 1‐dependent pathway, caspase 1 is activated by inflammasomes (NLRP1, NLRP3, NLRC4, AIM2 or pyrin) in the presence of the inflammasome adaptor protein, ASC. Caspase 1 then cleaves gasdermin D, which generates an N‐terminal fragment that induces cell death. In a caspase 1‐independent pathway, human caspase‐4/5 and mouse caspase‐11 can directly cleave gasdermin D to induce pyroptosis.^44^

Recent studies have also reported the role of pyroptosis in RPE cell death.^53^, ^55^, ^56^ Tseng and colleagues immunohistochemically detected the presence of NLRP3 inflammasome in areas of lesion in eyes affected by GA and CNV^55^. The presence of ROS is also shown to induce NLRP3 activation and pyroptosis in intestinal cells.^56^ Proteolytic cleavage of caspase 3 (apoptotic pathway) and gasdermin D (pyroptotic pathway) was seen in the RPE‐choroid tissues of rats receiving intravitreal injections of amyloid beta, a product of the complement cascade.^40^ The authors reported both apoptosis and pyroptosis being activated in these RPE cells. It is interesting to note that there was no thinning or loss of RPE cells during the morphological analysis in this study.^53^ When oxidative stress‐induced primary RPE was primed with inflammasomes, a switch in cell death mechanism from apoptosis to pyroptosis was also noted by Brandstetter et al.^57^

Although our understanding of the molecular mechanisms eliciting each cell death pathway has substantially increased, there is still debate about the mechanisms leading to RPE cell loss in AMD. It is difficult to distinguish between these cell death pathways, especially that of apoptosis and necrosis, as features such as chromatin degradation, DNA degradation and mitochondrial permeability are common to both.^44^ Additionally, it is also important to consider that if a particular cell death pathway is blocked, the mechanism might switch to another pathway, making it difficult to fully elucidate the originating pathway. However, further research will provide more insight into the mechanisms that are involved in AMD.

## 4 DYSFUNCTIONAL MITOCHONDRIA

Even though AMD is a multifactorial disease affecting different retinal cell types, recent years have seen the emerging theory that mitochondrial damage to RPE due to oxidative stress may be a reason for AMD disease pathogenesis.^58^, ^59^, ^60^ The mitochondria are primarily responsible for the energy demands of the cell and produce energy in the form of adenosine triphosphate (ATP) through oxidative phosphorylation (the primary ATP generation pathway), b‐oxidation and the citric acid cycle.^61^ RPE also metabolizes fatty acids to produce b‐hydroxybutarate, which can be used as an alternate energy source.^61^ Since the energy demands of the cell dictates the mitochondrion count, RPE cells have an enriched mitochondrial population to meet the energy demands of the outer retina.^62^

Early studies treating RPE cells with H_2_O_2_ to induce oxidative stress showed preferential damage of mitochondrial DNA and subsequently provided a rationale for a model of AMD that is mitochondrion‐based.^63^ Recently, there is increasing evidence of an age‐related decline in mitochondrial function in people with AMD. Mitochondrial numbers, area and density of mitochondrial matrix were shown to decrease with age along with partial‐to‐complete loss of mitochondrial cristae.^64^ Karunadharma et al reported significant damage to mitochondrial DNA (mtDNA) in subjects with AMD compared to age‐matched controls.^65^ Furthermore, Lin et al showed macula‐specific mtDNA damage increased with age.^66^ In RPE cells, one of the reasons that mtDNA damage could occur may be that an increase in ROS accumulation leads to an imbalance in homeostatic conditions of the retina and subsequent oxidative stress.^67^, ^68^, ^69^ However, ROS overproduction can also be a consequence of mtDNA damage.^70^

Sources of cellular ROS can be both mitochondria‐driven and non‐mitochondrial (eg, cigarette smoke). Mitochondrial generators of ROS include involvement of cytochrome P450, nicotinamide adenine dinucleotide phosphate (NADPH) oxidase (NOX), and xanthine oxidase (XO).^71^ However ROS is mainly produced as part of the mitochondria electron transport chain, which plays a vital role in intracellular signalling.^72^ In the cell, normal ROS levels are controlled and maintained by the anti‐oxidant system. However, when ROS levels surpass the antioxidant capacity of the cell, oxidative stress ensues.^71^ The exact mechanism of ROS build‐up in cells is unclear, however, some of the theories include a defective copy of the *SOD2* gene, exposure to visible light and lipofuscin accumulation.^52^, ^73^, ^74^

In the event of oxidative stress, *SOD2* scavenges for ROS thereby protecting the cell from potential damage.^75^ In a *SOD2* knockout mouse model, elevated levels of ROS and oxidative stress lead to mitochondrial alterations and RPE dysfunction when compared to wild type mice. Impaired ROS levels can also lead to mitochondrial dysfunction due to mtDNA damage.^76^

**FIGURE 2:**
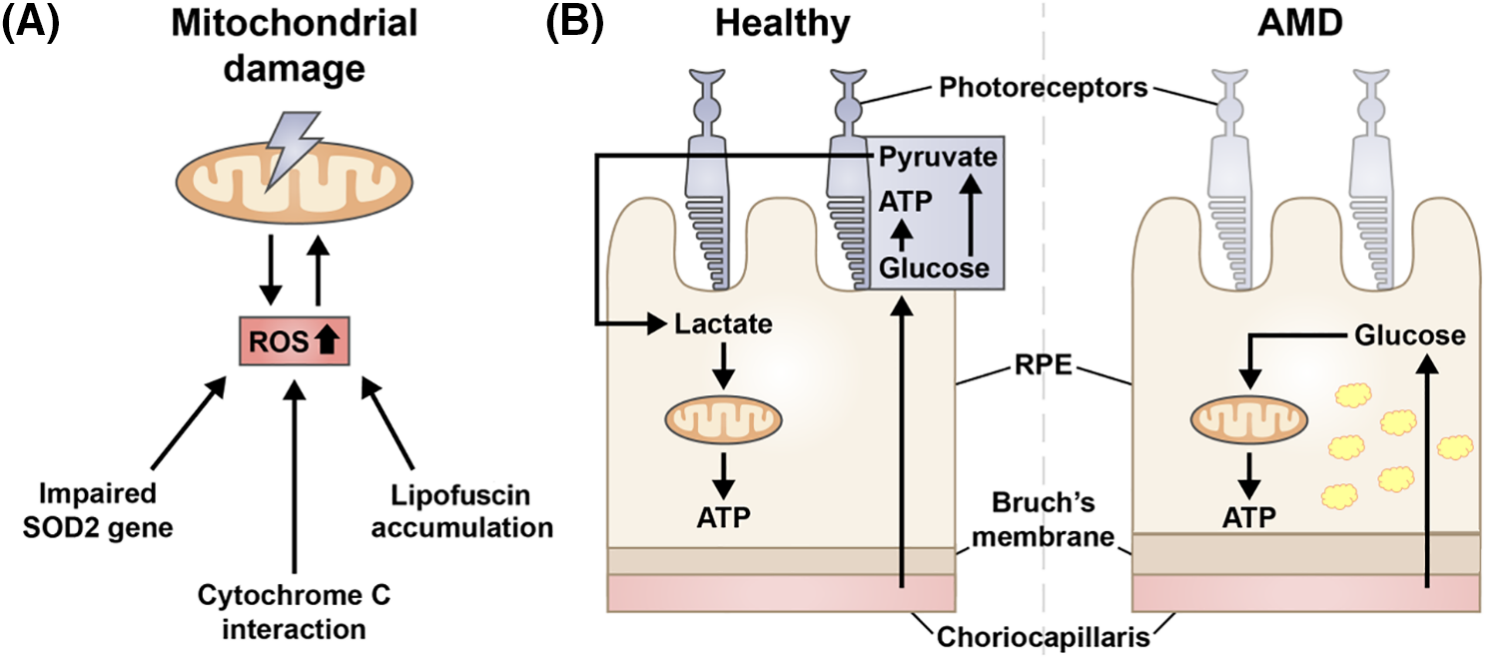
Mitochondria dysfunction in the retinal pigment epithelium (RPE) is another proposed mechanism in age‐related macular degeneration (AMD). Mitochondrial DNA damage is linked to an increase in reactive oxidation species (ROS) production. Although the causal relationship between these events is unclear, an impaired *superoxide dismutase 2* gene, cytochrome c interaction and lipofuscin accumulation can lead to increase in ROS production levels within the cell (A). Dysfunctional mitochondria due to metabolic coupling can also be a potential mechanism for AMD, where photoreceptor cell death ensues due starvation, as glucose meant to be used by the neural retina is consumed by the RPE (B). Note the thickening of the Bruch's membrane and accumulation of lipofuscin (yellow granules) within the RPE during AMD

ROS can also be generated through interactions of cytochrome c oxidase with the mitochondria. Transfer of energy from photoactivated chromophores to oxygen can lead to the formation of singlet oxygen which, through interactions with diatomic oxygen or electrons with double bonds, can lead to the generation of ROS.^77^ Accumulation of the age‐related pigment lipofuscin has also been shown to increase ROS levels within the RPE cells.^78^ Several mitochondrial proteins that are involved in the apoptosis cascade, such as cytochrome c and apoptosis‐inducing factor, can be released as a result of ROS overproduction and subsequent mtDNA damage.^79^ This can result in dysfunctional or damaged mitochondria, potentially leading to AMD pathogenesis (Figure 2A). Alternatively, RPE cells, although quiescent in the retina, could undergo oxidative stress‐induced senescence. Hydroxyl peroxide and cigarette smoke^80^ have been shown to induce senescence in RPE cells, which is proposed to play a role in the aetiology of AMD.^67^ A recent review by Correia‐Melo et al explores the idea of mitochondrial damage driving cellular‐senescence further.^81^ Based on the relationship between oxidative stress in retina and AMD, anti‐oxidants could be promising therapeutic agents. However, clinical trials by the Age‐Related Eye Disease Study (AREDS) investigating an anti‐oxidant formulation containing vitamins E and C, beta‐carotene and/or zinc as therapeutic supplements showed only a small, albeit significant, 10% reduction in developing advanced or neovascular AMD, but no significant reduction in developing central GA.^82^ Similarly, the use of other potent drugs with anti‐oxidant properties such α‐tocopherol and OT‐551 found no significant improvement.^83^, ^84^

A recent publication by Kanow et al, reviewed by Fisher et al, explored an alternate link between dysfunctional mitochondria and potential mechanisms for AMD through the idea of metabolic coupling between the neural retina and RPE, involving glyoclysis.^60^, ^85^ Glycolysis is the process of breakdown of glucose, producing energy in the form of ATP.^86^ The RPE transports the glucose received from the choroid to the photoreceptors. Photoreceptors utilize this glucose for glycolysis and, in turn, produce lactate. Lactate acts on the RPE to supress the use of glucose for energy production; instead, lactate is used by the RPE for energy production through oxidative phosphorylation. In AMD, when mitochondrial function is disrupted, RPE mitochondrion consume the glucose and rely on glycolysis to produce ATP, reducing the available glucose to photoreceptors, thereby starving the neural retina and causing cell death^60^, ^85^ (Figure 2B). A study by Golestaneh et al supported this theory by showing that the RPE of patients with AMD relied on glycolysis as the primary source of ATP rather than oxidative phosphorylation.^87^ However slightly conflicting results from another study shows that ATP production via oxidative phosphorylation and glycolysis is decreased in primary RPE from patients with AMD.^88^ Although the connection between mtDNA damage, ROS overproduction and RPE dysfunction is clear, the exact mechanism regarding the onset and progression of AMD still needs further work and evaluation.

## 5 AUTOPHAGY

Autophagy is a basic housekeeping process that involves a lysosomal clearance of damaged and dysfunctional proteins and organelles in order to maintain cellular homeostasis.^89^, ^90^ Although there are three different autophagic pathways (macroautophagy, microautophagy and chaperone‐mediated autophagy), it is macroautophagy (hereafter referred to as autophagy) that is primarily involved in mammalian cells.^90^

The promoter, AMP‐activated protein kinase (AMPK), and the inhibitor, mammalian target of rapamycin (mTOR), are two critical regulators of this process. Activation of AMPK or inhibition of mTOR initiates autophagy. The process begins with the formation of the phagophore that originates from the endoplasmic reticulum. The phagophore elongates and ingests the degraded protein/organelles (cargo) to form the double‐membrane autophagosome. Several autophagy‐related proteins (ATGs) along with the microtubule‐associated protein 1A/1B‐light chain 3 (LC3) conjugation system facilitate autophagosome maturation.^90^, ^91^ Upon maturation, the autophagosome fuses with the lysosome to form the autolysosome, where the contained waste is degraded.^90^, ^91^ RPE cells usually undergo basal autophagy to maintain cellular homeostasis in the retina.^89^ Studies have shown that there is a significant increase in LC3, ATG7 and ATG9 in RPE of aged non‐AMD patients,^92^ suggesting an increased burden on the autophagic process to clear out damaged organelles as ageing occurs.

The exact role of autophagy in AMD is still unknown. However, impaired lysosomal degradation due to accumulation of lipofuscin was one of the earliest hypotheses put forward.^89^ Cathepsins are lysosomal proteases that degrade proteins and are found in the RPE with the major task of degrading POS. When POS degradation reduces, intracellular RPE stress occurs due to the formation of metabolites such as lipid peroxidation end products and oxidized low‐density lipoproteins.^91^ The formation and storage of these metabolites begins the process of lipofuscinogenesis.^93^ Once lipofuscin is formed, it cannot be degraded by lysosomal enzymes. Additionally, lipofuscin can increase oxidative stress in the RPE which, upon light exposure can further reduce lysosomal cathepsin activity. This could lead to an accumulation of autolysosomes, with high levels of partially degraded material, causing drusen.^89^, ^93^ Interestingly, Wang et al has noted the presence of autophagy markers in drusen obtained from AMD donor tissue.^94^

There has been mounting evidence to suggest that autophagy in the RPE decreases due to chronic exposure of RPE to mitochondrial oxidative stress.^91^, ^92^, ^95^ Mitter et al reported a dramatic decrease in autophagy when RPE cells were chronically subject to oxidative stress through H_2_O_2_ exposure. On the other hand, they also reported an increase in autophagic flux when RPE cells were subjected to acute oxidative stress. They concluded that autophagy is usually increased in early AMD to compensate for the increase in oxidative stress on the RPE, however, by later stages of AMD, the autophagy process is unable to cope with the increased amount of damaged organelles and thus becomes impaired. They were also able to show that stimulation of autophagy through rapamycin protects RPE cells from mitochondrial oxidative stress, and inhibition of autophagy causes mitochondrial activity and cell viability to be compromised.^92^

Potential therapeutic strategies involving upregulation of autophagy may prove to be useful. Yet targeting the signalling pathways in autophagy may be difficult, as it is a fundamental housekeeping process in many cell types. Additionally, accumulation of lysosomal lipofuscin could limit the regulation of autophagic flux. Although the exact mechanism by which autophagy affects AMD is unclear, there is sufficient evidence to suggest further research into this area could prove to be beneficial.

## 6 THE ROLE OF ΑB CRYSTALLIN IN AMD

Small heat shock proteins (sHSP) help with the assembly of cellular proteins, and act to guide incorrect or misfolded proteins. They are activated with a protective function of preventing proteins from denaturing when a cell undergoes external stress.^96^ sHSP have shown to be involved in the apoptosis cascade, with some sHSPs acting in an anti‐apoptotic manner, while others are pro‐apoptotic.^97^ α‐crystallins are an important member of the sHSP family and are expressed in the cellular cytosol and mitochondria. αA and αB crystallins are the major members of this family. αA crystallin is found mostly in the photoreceptors, astroglia and Müller glia cell populations, whereas αB crystallin is found mainly in the RPE, localized to the mitochondria and golgi apparatus. This suggests that αB crystallin might play a role in golgi reorganization during the cell cycle.^98^

Several studies have studied the expression of αB crystallin in diseases including AMD.^96^, ^99^, ^100^ In a study by Soma De et al, a significant increase in expression of αB crystallin was found in cultured RPE from donors with advanced AMD, when compared to donors with earlier stages of AMD or age‐matched controls.^100^ Similarly, higher αB crystallin expression was noted near hypertrophic regions and drusen‐associated regions of the retina in patients with neovascular AMD and early atrophic AMD, respectively.^100^ The expression of this protein was noted to further increase under stressed conditions, with αB crystallin expression levels significantly higher when RPE was subjected to oxidative stress by H_2_O_2._ ^96^ Further, high‐stress areas of the retina, such as the macula, seem to have higher expression levels of αB crystallin than peripheral regions.^96^ When compared to baseline levels, high levels of αB crystallin were also detected at the RPE‐choriocapillaris interface and in Bruch's membrane of donors with AMD. This is interesting, as Bruch's membrane is acellular, implying that the protein either leaked from the adjacent RPE or other tissues, and its presence in this space could suggest that it was there to repair, or, alternatively, it was there to chaperone the damaged proteins.^101^

It was reported that αB crystallin acts as a chaperone for VEGF and protects the protein against aggregation and unfolding under conditions of stress.^97^, ^102^ This is potentially detrimental, as neovascular AMD (CNV) arises due to VEGF overproduction by the RPE. This observation is consistent with other studies that show an upregulation of αB crystallin expression with angiogenesis^103^ and significantly lower VEGFA protein levels in αB crystallin knockout mice.^104^ A study by Kase et al has also shown that the size of the laser induced CNV lesion in crystallin knockout mice was significantly smaller when compared to wild type.^104^ However, it is unclear whether this protein acts directly on the RPE or vascular endothelial cells. Although αB crystallin is suggested to play an important role in the development of retinal vasculature^104^ and the intracrine stimulation of VEGF by endothelial cells,^105^ in vivo studies exploring retinal vasculature in a αB crystallin knockout mouse model did not show major vascular defects.^105^ This warrants further research to fully understand the role and mechanism of αB crystallin in angiogenesis.

**FIGURE 3:**
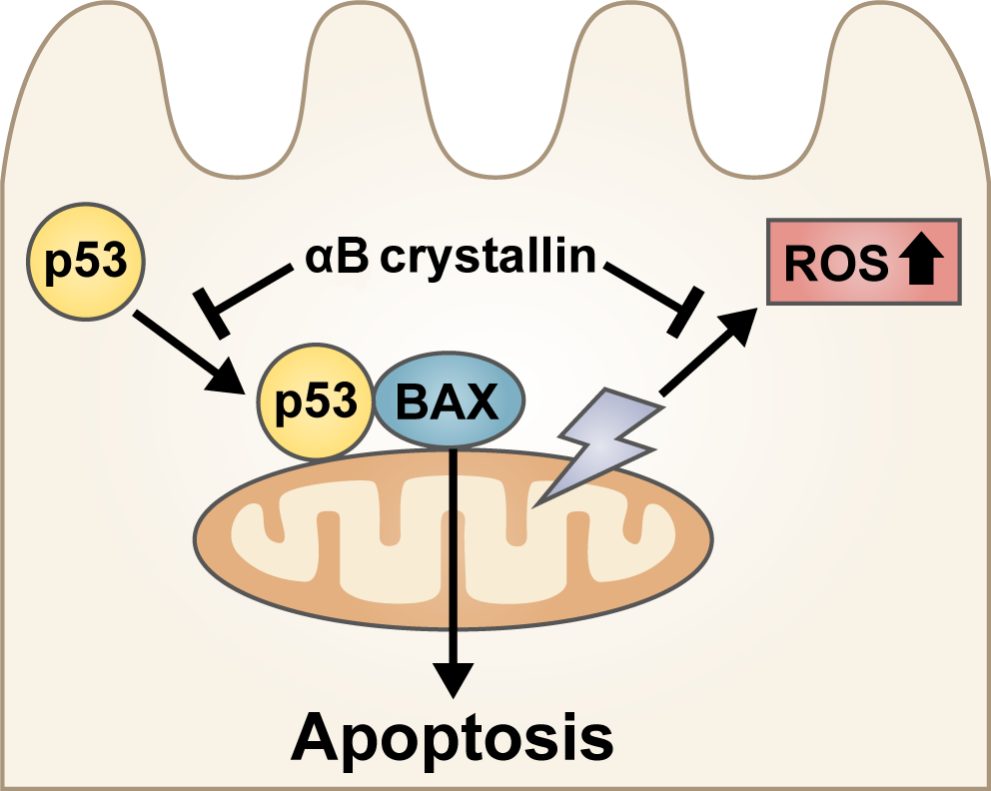
αB crystallin may confer a protective role in the pathogenesis of age‐related macular degeneration (AMD). αB crystallin could potentially inhibit reactive oxygen species (ROS) overexpression and apoptosis driven by p53 translocation to the mitochondria

On the contrary, studies also show that αB crystallin might serve a protective function in the retina (Figure 3). In 2002, one of the first studies looking at the role of αB crystallin in AMD showed that αB crystallin may protect RPE cells from an apoptotic cell death in response to oxidative stress.^106^ Similarly, αB crystalline‐transfected RPE cultures were found to be more resistant to oxidative stress‐mediated injury^96^, ^97^, ^107^ and an increase in apoptotic activity was noted in a crystallin knockout mice model,^99^ where CoCl_2−_induced oxidative stress led to severe and rapid retinal degeneration.^99^ It was also noted that αB crystalline‐positive RPE cells did not undergo cell death via apoptosis.^100^ As discussed, cell death via apoptosis is mediated via two pathways; the mitochondrial/intrinsic (cytochrome c dependant) pathway and death receptor mediated/extrinsic (capase‐8‐dependent) pathway, where both pathways lead to the activation of caspase 3, which heralds apoptosis of the cell. In the intrinsic pathway, p53 translocates from the cytoplasm to the mitochondria to upregulate Bax expression, which triggers the apoptosis cascade.^43^ Watanabe et al reported that αB crystallin could possibly inhibit apoptosis driven by the intrinsic pathway by interacting with p53 to prevent its translocation to the mitochondria.^108^ αB overexpression also blocks ROS activation to inhibit apoptosis through the intrinsic extracellular receptor kinase 1 and 2 (ERK1/2) pathway.^109^

While there is no direct evidence indicating the role of endoplasmic reticulum (ER) stress in AMD, the cross‐talk mediated by stressor signals between the ER and mitochondria may lead to mitochondrial dysfunction and apoptosis. Increased apoptosis was found in RPE cells isolated from αB crystallin knockout mice following prolonged ER stress, suggesting that αB crystallin might be protective in ER stress‐induced apoptosis.^102^ The therapeutic benefit of crystallins has also been explored in a study by Sreekumar et al. αB crystallin is too long to be taken up by RPE cells, but delivering short 19‐mer peptides of αB crystallin to human fetal RPE has revealed that the two Na^+^coupled transport systems, sodium‐coupled oligopeptide transport system 1 and 2 (SOPT1 and SOPT2), can mediate the uptake of the mini‐chaperones into RPE cells. Once inside the cell, these mini chaperones seemed to have an anti‐apoptotic effect by protecting the RPE from H_2_O_2_‐induced cell death through inhibition of caspase 3 activation.^110^

This paradox of both a protective and detrimental role for αB crystallins poses a research challenge. Furthermore, there is a lack of literature illustrating the mechanisms of action of αB crystallin in AMD. Nonetheless, it is clear that increased expression of αB crystallins could serve as a biomarker for AMD. Further research exploring a potential link between crystallins and dysfunctional mitochondria and elucidation of the mechanisms of action of αB crystallins in the progression of AMD is warranted to evaluate this paradox.

## 7 FUTURE DIRECTIONS

There are many animal models of AMD that mimic the pathological features commonly seen in the disease, including mouse models with oxidative damage and laser induced CNV models.^111^ However, no one model recapitulates all of the features observed in AMD. Additionally, the complex and heterogeneous nature of AMD and the phenotypic diversity encountered in the disease makes developing a comprehensive animal model extremely difficult. More recently, RPE‐based models, including cultured RPE cells, have gained a lot of momentum and have been promising in the investigation of molecular mechanisms of AMD.^112^

In order to better understand disease mechanisms, culturing primary RPE is ideal; however these cells are usually obtained post mortem and are often complicated by secondary disease mechanisms, coupled with technical differences between sample collections. Immortal cell lines such as ARPE19 offer an alternative, although immortalized lines may not completely emulate the physiology of primary RPE.^113^ Gene therapy has shown promising results, with AAV‐delivered therapeutics being FDA approved for several ocular conditions, however this would be a difficult approach given the complexity of AMD pathogenesis.^39^ Research into AMD through the use of human induced pluripotent stem cells has shown great possibility as a platform for understanding underlying pathology and testing out therapeutic approaches.^114^ With the advent of efficient protocols for the differentiation of stem cells into RPE^86^ and increasing evidence that stem cell‐derived RPE shares many characteristic features of native RPE,^115^ there are increasing opportunities to test this approach in the field of personalized medicine.

## 8 CONCLUSION

The identification of underlying disease mechanisms is crucial to the understanding and treatment of any disease. There are several factors and mechanisms that influence the origin and progression of AMD, making it a challenging disease to study and treat. However, recent studies have made huge progress in understanding alternate disease pathways, as outlined in this review, widening the scope for treatment development. Transplantation of RPE cells to replace degenerated RPE is an alluring possibility, but will likely be limited to patients with intact photoreceptors or minimal photoreceptor atrophy.^116^ Clinical trials to modulate the complement system have had minimal therapeutic benefits.^38^, ^39^ It is also likely that several widely accepted disease causing factors such as smoking and a western diet high in saturated fats could still lead to disease progression in those who are genetically predisposed.^117^ Since AMD is a multifactorial disease, with potentially more than one pathological mechanism, it is likely that intervention may need to be multifaceted. Although the precise RPE‐related disease mechanisms in AMD are yet to be fully elucidated, there is now substantial insight into a range of probable contributing factors. These can form the basis of translational projects to develop novel therapeutic remedies for AMD.

**TABLE 1:** Glossary of common terms used in this review

Abbreviations	Full name	Function
MAC	Membrane‐associate complex	An effector protein of the innate immune system that forms cytotoxic pores on the cell surface of pathogens
CFB/F/H	Complement factor B/F/H	Regulator proteins of the complement activation cascade
SERPING1	Serine peptidase inhibitor clade G member 1	The SERPING1 gene encodes the protein C1‐inhibitor, a protease inhibitor
HTRA1	HtrA serine peptidase 1	The HTRA1 gene encodes the protein serine protease, which cleaves peptide bonds
TNF	Tumour necrosis factor	A member of the group of cytokines that activates an acute phase reaction in systemic inflammation
RIPK	Receptor interacting protein kinases	Key sensors of intracellular/extracellular stress and regulators of cell death/survival, found in the necrosis cell death pathway
ROS	Reactive oxidation species	A byproduct of oxygen metabolism that plays a key role cell signalling and homeostasis
Bax	Bcl‐2‐associated X protein	Bax is a protein that heterodimerizes to function as an apoptotic activator
SOD2	Superoxide dismutase 2	SOD2 protects cells against cell death by clearing excess mitochondrial ROS
NLRP3	Nod‐like receptor family pyrin domain containing 3	A scaffolding protein that assists with the formation of inflammasome, found in pyroptosis
ATP	Adenosine triphosphate	ATP is the source of energy for many cellular processes and is generated in the mitochondria as a result of glycolysis, the citric acid cycle or the electron transport chain
NAPDH	Nicotinamide adenine dinucleotide phosphate	NAPDH is a cofactor of anabolic reactions and functions as the electron donor/reducing agent

## FINANCIAL DISCLOSURE

This work was funded by the Lions Eye Institute (LEI ‐ Livia S Carvalho) and a LEI/Lions Save Sight Foundation (LSSF)‐funded Brian King Fellowship (Carla B. Mellough).
